# Topical and emotional expressions regarding extreme weather disasters on social media: a comparison of posts from official media and the public

**DOI:** 10.1057/s41599-022-01457-1

**Published:** 2022-11-28

**Authors:** Ziqiang Han, Mengfan Shen, Hongbing Liu, Yifan Peng

**Affiliations:** 1 School of Political Science and Public Administration, Shandong University, Qingdao, China; 2 Center for Crisis Management Research, Tsinghua University, Beijing, China; 3 School of Information Science and Engineering, Shandong University, Qingdao, China; 4 Department of Population Health Sciences, Weill Cornell Medicine, University of Cornell, New York, NY USA

**Keywords:** Environmental studies, Cultural and media studies, Psychology

## Abstract

Understanding media frames and the public resonance during disasters is essential for making inclusive climate change and adaptation policies in the context of increasingly extreme weather events. In this study, we use the extreme weather and flood event that occurred in July 2021 in Zhengzhou, China, as a case study to investigate how official media in China reported this event and how the public responded. Moreover, since one accountability investigation report regarding this disaster was released in January 2022, we also compared these posts between the emergency response period and the post-crisis learning period after the report’s release. Topic modeling using the LDA (Latent Dirichlet Allocation) method and emotion analysis were conducted to analyze the posts from Weibo, China’s primary social media platform. The results demonstrated that the posts from official media and the public comments differed in both topics and emotions, with relatively little coherence. During the emergency response period, the media’s posts focused more on the facts, such as the extreme weather event, the places where it occurred, the impacts, and the search and rescue efforts, while the public comments were more about help appeals from the neglected ones in the rural areas, and emotional expressions such as moral support, condolence or encouragement to the victims and their families. After the accountability investigation in January, the media’s posts primarily covered the investigation process, the punishment, the attribution of disaster consequences, and the lessons learned, while the public’s comments were relatively emotional, praised the good, condoled the victims, and condemned the villains. The dominant emotion from the media’s posts was “like” in July 2021, but it became depression in January 2022. Anger was the prevalent emotion from the public during all the stages. This study provided valuable knowledge to the current understanding of the different patterns and dynamics of official media reports and the public’s resonance in disaster management.

## Introduction

According to a new report from the United Nations, there has been an increasing trend of extreme weather events globally, from 3656 climate-related disasters in the period 1980–1999 to 6681 events between 2000 and 2019 (CRED & UNDRR, 2020). For example, 20.96% and 19.60% of the U.S. land area experienced extreme one-day precipitation in 2015 and 2017 (Ritchie & Roser, 2022). Moreover, extreme weather events have been seen globally in the past year, from Europe to Asia, America, and the Ocean Continent. Two hundred thirty-nine people lost their lives during a flood in Belgium and western Germany in mid-July 2021 (Wikipedia, 2022b), 398 residents perished in a flood in Henan province, China, in late July (Wikipedia, 2022c), and Hurricane Ida in August claimed 87 lives in the United States (Wikipedia, 2022a). In March 2022, a flood in Australia also caused at least 22 deaths in Queensland and New South Wales (Wikipedia, 2022d). These disasters derived from extreme weather events have gained lots of media, public, and policy makers’ attention, especially in the context of the United Nations Climate Change Conference—the COP 26 (the 26th session of the Conference of the Parties) with a purpose of designing the next critical steps in mitigating climate change among global members. Studies indicated more extreme weather events in the coming years (Nullis, 2021). Better policy and adaptation can be developed only by integrating the public’s opinions and lessons learned from past events. Therefore, it is crucial to understand the media’s framing and public perceptions of these extreme weather events and the disasters they induced because they are critical for agenda-setting in policymaking and policy changes (Albright, 2020; Birkland, 2016).

Social media has become a primary Information Communication Technology (ICT) employed by the general public and institutions, especially in disaster and emergency management situations (Abedin & Babar, 2018; Behl et al., 2021; Vo & Collier, 2013). The citizens and authorities, also termed the general public and organizations, are the two perspectives primarily considered in social media and disaster studies (Reuter & Kaufhold, 2018). During emergencies or disasters, the general public usually uses social media as a primary source of seeking information, communicating with others, expressing their emotions, help-seeking, or organizing for self-help (Reuter & Kaufhold, 2018; Silver & Matthews, 2017). Emergency managers or government agencies use social media as a critical channel for risk and crisis communication with the public. Meanwhile, the emergency professionals may also use information from social media platforms for collective intelligence activities such as quick impacts assessment (Fang et al., 2019; Wu & Cui, 2018), detecting public sentiments (Iglesias-Sánchez et al., 2020) or situation awareness (Karimiziarani et al., 2022), and such information can help them make critical decisions for emergency response. Moreover, some organizations, especially non-government organizations or self-organized groups involved in disaster response, may use social media for response coordination (Reuter & Kaufhold, 2018).

Last but not least, discourse on social media can be a vital source for understanding people’s understanding, attribution, and cognization of climate change, extreme weather, and the relevant adaptation strategies and policy preferences (Houser et al., 2022; Howe, 2021; Ogunbode et al., 2019; Roxburgh et al., 2019). Therefore, social media has become a crucial arena for almost all actors involved in disaster and emergency management. Thus, investigating the information generating and spreading patterns on social media can contribute to emergency management studies and the psychological or behavioral sciences in cyberspace.

Even the traditional mass media use social media as an essential method of engaging with and communicating with more audiences nowadays, especially for the young generations (Tsuriel et al., 2021). In the past, mass media play a significant role in delivering beliefs and human behaviors about disasters, especially before the emergence of social media. However, the mass media tend to exaggerate the incidence of antisocial behavior such as looting and lawlessness instead of the dominant prosocial behavior (Rodríguez et al., 2006). The disaster myths framed by mass media can reinforce the calling for a greater role of social control in disaster response (Tierney et al., 2006), which is not an ideal disaster management model in modern societies (Waugh & Streib, 2006). Meanwhile, since disaster can trigger a potential window for policy change (Birkland, 1997), the discourses on policy issues, accountability, and responsibility attributions can be the general coverage in media discussions that go beyond the realm of an incident. For example, after Hurricane Maria in Puerto Rico, the New York Times and Wall Street Journal portrayed the disaster as a poor financial and governance capacity of Puerto Rico instead of the inadequate response of FEMA and the federal government (Straub, 2021). When media align with the government or a particular political side, the narrative of an event could be softer or primarily on the “natural” hazards aspect, that means the flood, earthquake, etc., instead of the disaster aspect, which includes the societal roots of vulnerability. In these cases, the media’s frames of disaster response can also emphasize the government’s triumph or the people’s solidarity (Liu & Boin, 2020; Saldaña, 2022). However, it should be noticed that even in a society where the state can heavily influence media, the different media can demonstrate varied opinions regarding controversial issues (Du & Han, 2020), and subtle differences can still be detected through appropriate analysis methods. Therefore, analyzing the media frame regarding disasters, especially extreme events, is critical to understanding the various stakeholders’ opinions and policy preferences.

Media frames can influence the public’s opinions and attitudes. However, the linkage between media frames and the public’s resonance is rarely examined, though the dynamics and interactions between political leaders and their followers are very well studied in the political science field (Haman, 2020). Risk and crisis communications from authoritative sources are important for disaster and emergency management, especially in the context of misinformation and disinformation widely available online. Unfortunately, the dynamics between institutions or media posts and the general public’s resonance is not well investigated besides in the recent Covid-19 risk communication scenarios. The public’s responses to Canada’s Chief Public Health Officers’ posts on Twitter and YouTube were quite different—Twitter comments were more balanced in tone and emotions, but YouTube comments were more sarcastic (Hodson et al., 2021). Capturing the interaction patterns between official posts and the general public can contribute to the current understanding of human behavior in cyberspace, improve risk and crisis communication efficacy, and eventually facilitate disaster and emergency management practices.

Therefore, we investigated the dynamics and interactions between the public and the official media using the 2021 summer flood in China as a case to understand the variety of perceptions between the official media and the public. We use the term “official media” because all the mass media press in China are state-owned. We used the posts from these official media accounts on social media (Weibo) instead of the traditional outlet because social media is the main place to capture the interactions between the media’s posts and the public’s resonance in China. Most mass media website closed their comments from readers. It should be noticed that the mass media in China, especially those associated with state agencies, play dual roles as relatively independent media and the voice of government and the ruling Communist Party of China (CPC). Thus, the official media’s posts on Weibo (social media platform) and the public comments under these posts is the main place to see the dynamics and interactions between official media and the public’s resonance.

Therefore, we first identified the official media’s Weibo accounts related to various central government agencies and then collected all their posts on Weibo regarding the 2021 summer floods that occurred in Henan province from July 2021 to March 2022, as well as the public’s comments underneath their posts. Using the topic modeling and emotion analysis, we will answer the following research questions in this paper.

RQ1: What are the topics and emotional expressions in the posts from official media and the public’s comments during the emergency relief period?

RQ2: What are the topics and emotional expressions in the posts from official media and the public’s comments during the crisis learning period?

RQ3: How did the topics and emotional expressions in the posts from official media and the public’s comments and their difference change between the two periods?

## Methods

### Background

A massive flood struck Zhengzhou—the biggest city of Henan province with a population of about 12.6 million July 20, 2021, following extremely heavy precipitation that started on July 17. The flood caused 380 deaths and 40.9 billion (RMB) economic losses within the city, while 398 deaths and 120 billion economic losses in total in this region, according to the final accountability investigation report released by the central government of China. Since this extreme weather event caused such massive casualties in a big city, an accountability investigation was initiated by the central government of China to find out the vulnerability and reasons for such loss. The Ministry of Emergency Management was designed as the leading agency, with a group of experts and professionals from the relevant government agencies and research institutions. Six months later, the central government released the investigation report on January 21, 2022, and the report demonstrated that the disaster was not a purely “natural” disaster but a consequence of poor mitigation and preparedness efforts in the city, as well as the local government’s poor performance during the response period. Ninety-seven local officials and managers from the state-owned utility companies, including the city mayor, were under penalty. Some infrastructure contractors were also arrested for further criminal investigation and prosecution. Since this is a rare disaster that hit large cities and caused such enormous consequences, studies based on this case should be valuable for future disaster and emergency management, especially in the context of climate change and the increasing likelihood of more extreme weather events.

### Data collection

**Fig. 1:**
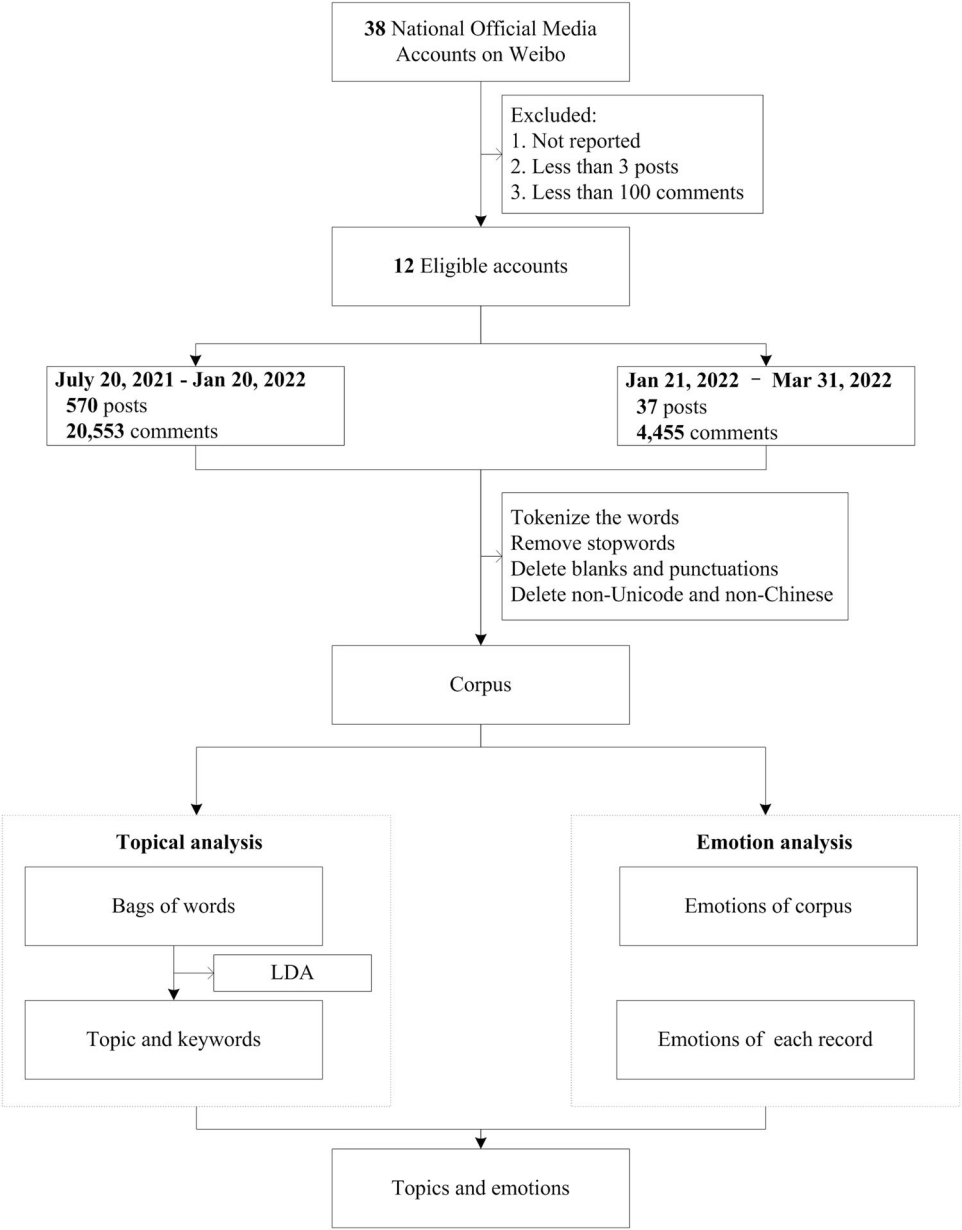
Data collection and processing flowchart.

We collected the posts from official media agencies and the public’s comments on the largest social media platform in China, Weibo. According to the Regulation of News Service on the Internet, thirty-eight national official media agencies can produce and disseminate news in cyberspace from the Cyberspace Administration Agency of China (Cyberspace Administration of China, 2021). As shown in Fig. 1, we first identified the accounts of the 38 national official media and then searched their posts regarding the 2021 summer flood in Zhengzhou from July 10, 2020, to March 31, 2022.We set the March 31, 2022 as the last day of data collection because we finished this paper in April. We found that 20 media reported the event. We then screened the accounts with at least three posts regarding the event or posts with more than 100 comments.

Both the posts from official media accounts on Weibo and the public comments regarding these posts were collected for analysis. We manually collected the posts posted by official media, and then crawled the public comments under these posts by the code-based method. We used January 21, 2022, as the cut point between the crisis response and crisis learning stages because it was the day when the accountability investigation report from the central government was released. Between July 20, 2021, and January 20, 2022, 570 posts regarding the flood from 12 official media accounts and 20,553 public comments were identified under these posts. After releasing the accountability investigation report, 37 posts from the official media and 4455 comments from the public were identified.

We collected the official media agencies’ posts on social media platforms instead of their reports for three reasons. First, social media has become the primary information outlet for many organizations, including the traditional mass media. Mass media agencies usually have social media editors to extend their presence and reach more people on social media (Tsuriel et al., 2021). Second, the public and netizens would post comments or forward or reply to the posts of the traditional mass media on the social media platform. Thus, social media can capture the interactions and communication dynamics between the public and the traditional media. Third, social media is the most critical information-seeking channel for most of the general public during emergencies. Therefore, they would double-check the information about an emergency from the social media accounts of government agencies or reliable mass media for verification (Wu et al., 2018).

### Data analysis

**Fig. 2:**
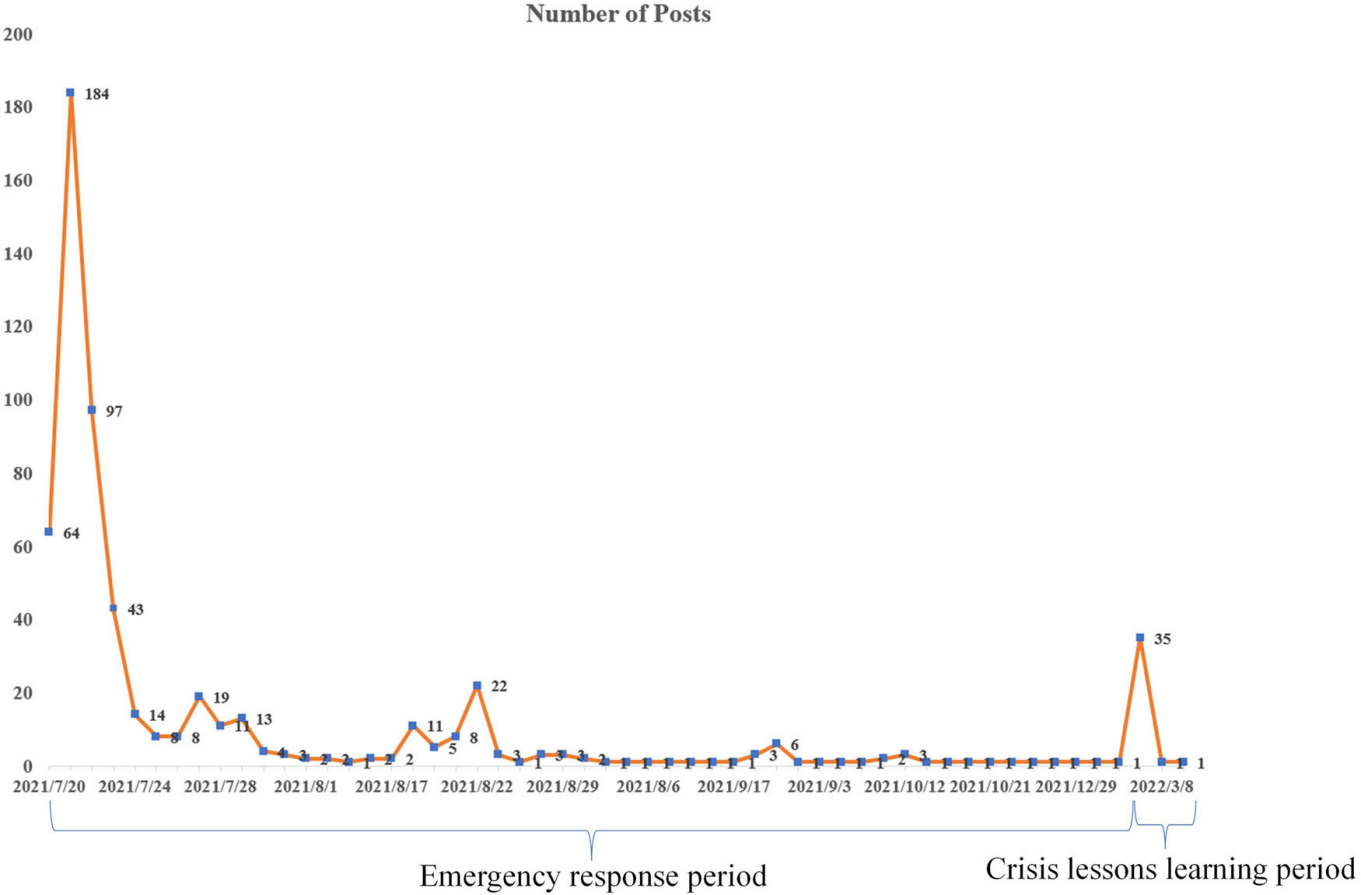
Posts from official media by the time.

We analyzed two types of content in this study. The first was the Weibo posts from the official media accounts. The second was the public’s comments following these posts. As shown in Fig. 2, there were two concentrated periods regarding this event from the media posts. The first period started from the event’s occurrence and primarily concentrated within 1 month after the event, and we defined this period until the release of the investigation report as the “emergency response period.” The second period covered another month after the release of the accountability investigation on January 21, 2022, and we defined this period as the “crisis lessons learning period.”

After collecting the text data, we first applied the regular expressions to detect the non-Unicode characters, blanks, punctuation, and non-Chinese phrases. These characters were removed from the analysis. We also removed duplicated sentence expressions using the mechanical compression method (Zhang, 2016). Finally, we used the Jieba, a widely used Chinese text segmentation, to tokenize the words and remove the stop-words (Peng et al., 2015).

The Latent Dirichlet Allocation (LDA) method was employed to conduct the topical analysis of the posts from the official media and the comments generated by the public, respectively (Blei et al., 2003). We chose the LDA method for topic modeling for two reasons. First, LDA is one of the most popular topic modeling methods that can discover hidden themes (topics) in a collection of documents in an unsupervised way. Second, Chinese text is denser than English; the corpora to be analyzed are the integrated corpora of all posts and comments in each period. Therefore, though the LDA method has limited application to short text (less than 140 characters) (Chen et al., 2013; Hong & Davison, 2010; Quercia et al., 2012; Ramage et al., 2010), the combined posts and comments were long text, and thus the LDA method is appropriate. In our case, LDA posits that each corpus was a mixture of a small number of topics and that each word was attributable to one of the topics.

To train the LDA model, we first constructed bag-of-words for each post, i.e., vectors consisting of words and their frequencies, as the input for the LDA model. We then used the gensim library implementation of LDA to extract topics and keywords (Řehuřek & Sojka, 2011). For LDA, we need to specify in advance the number of topics (*k*) in the underlying topic structure. To find the optimal *k*, we compared perplexity (how well a model predicts a sample) across different models with varying *k*s. The model with the “lowest” perplexity is generally considered the “Robust” model. We plotted the variation of perplexity with the number of topics. The result showed that the minimal perplexity value appeared when the number of topics was 5. Table 1 shows the topics and top-10 keywords in each topic.

**Table 1:** Topics and keywords during the emergency response period (07/20/2021–01/20/2022).

	Topic	Keywords	%
Media posts	Hazard	Storm, extreme, heavy rain, precipitation, disaster, flash flood, warning, covid-19, weather, Henan, Zhengzhou	30.8%
	Location	Metro, Line 5, Zhengzhou, between stations, train platform, community, Henan, hospital, weather station, Northern region	19.8%
	Rescue	Hotel, rescue, vehicle, firefighter, search, flood prevention, volunteer, trapped, escaped, hospital	17.1%
	Metro	Rain, metro, tunnel, rescue, trapped, line 5, in operation, driver, lost, passenger	16.8%
	Impacts	Dead, statistic, emergency, recuse, stuck, recovery, perish, firefighting, stress, trapped	15.4%
Public comments	Help-seeking	Tear, candle, kneel, Xinxiang, search, rescue, metro, Gongyi, help, Weihui, trapped, casualties	24.0%
	Moral support	Peace, hold on, mutual assistance, rescue, hard work, expect, victim, Zhengzhou, hometown	21.7%
	Donation	Add oil, donation, red cross, foundation, charity, bless, confidence, trust, channels	18.6%
	Condolence	Perished, peace, hero, salute, life, disaster, heart breaking, flower, peace, good-heart	18.1%
	Places-neglected	Weihui, Henan, Xinxiang, metro, railway, Gongyi, China, western road, township, village	17.6%

**Fig. 3:**
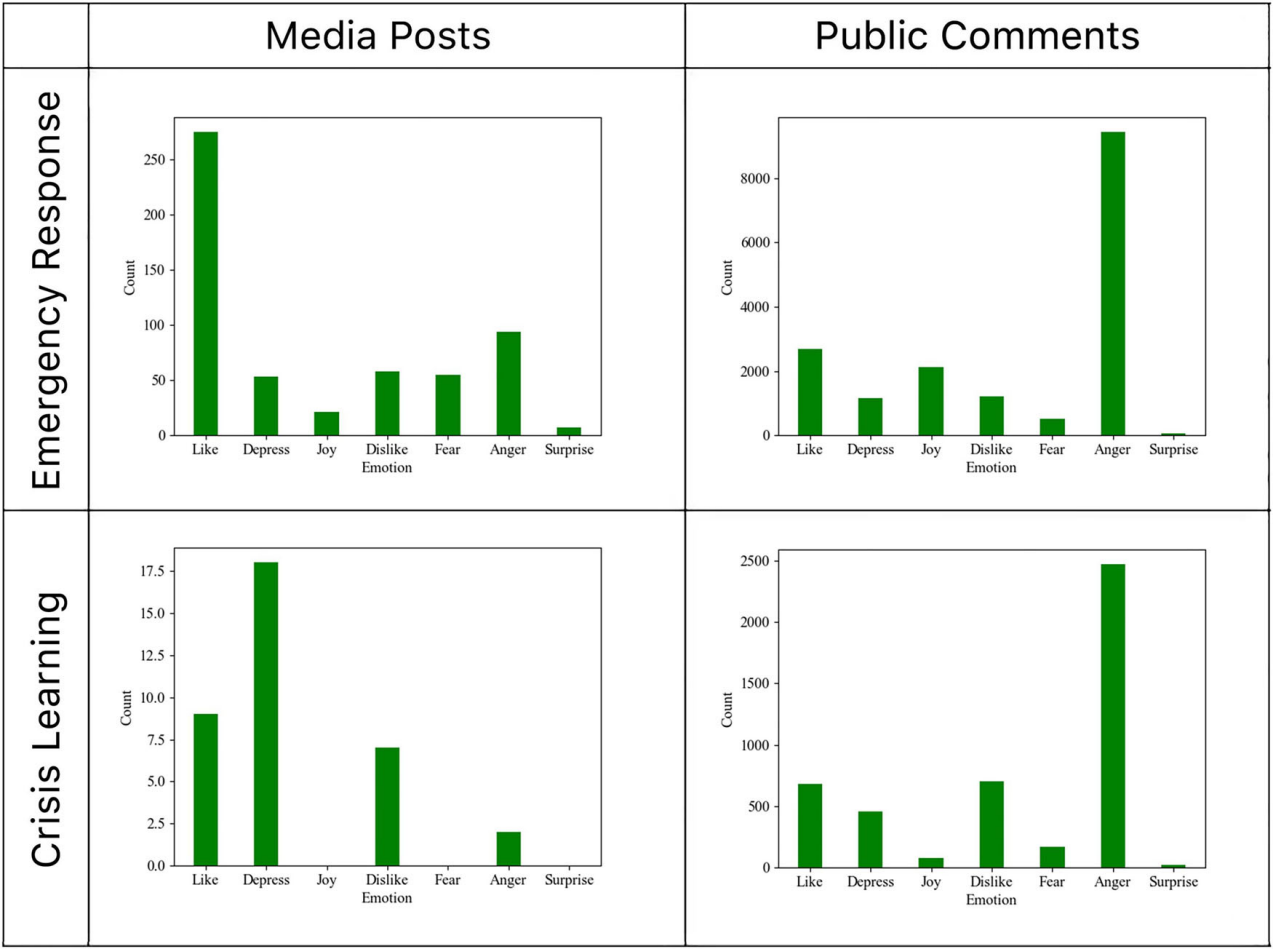
Emotions from the media and public posts.

We used the DUTIR codebook of emotions for the sentimental analysis (Xu et al., 2008). This codebook includes 27,466 Chinese emotion words and divides them into seven major categories (depression, like, dislike, fear, surprise, joy, and anger) and 21 subcategories, as shown in the supplementary Table 1. We counted the frequencies of each DUTIR emotion word in our corpus and aggregated them according to the DUTIR major categories. Figure 3 shows the different distributions between the media and public posts in two periods.

**Fig. 4:**
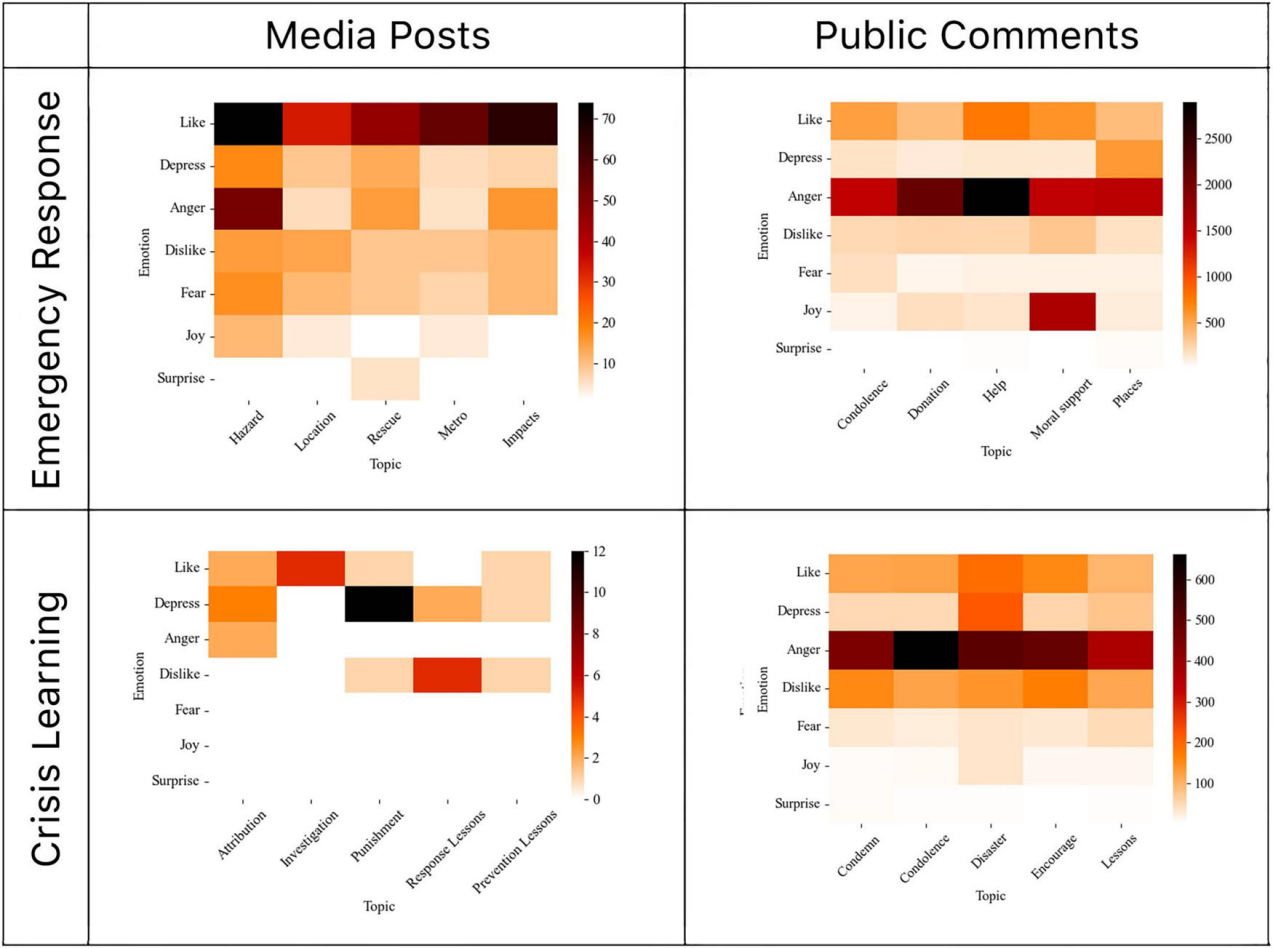
Emotions by topics.

Then we tabulated the emotions by topics in Fig. 4. The emotion of each corpus was coded by the emotion words with the highest frequency. The output was used as the column of data. Then, we traversed each corpus and ran the LDA model to derive the topics, keywords, and probability distribution. Finally, a heat map of the sentiment distribution by topics was generated (Fig. 4).

Moreover, since the posts and comments were concentrated during the two periods, we reported the topics and emotions by the types of generators (official media’s posts or the public’s comments) and compared the topics and emotions between the two periods.

## Results

During the emergency response period, from July 20, 2021, to January 20, 2022, five topics emerged from the official media posts: hazard, location, metro, rescue, and impacts (Table 1). The hazard topic (30.8%) primarily covered the description of the rain and flood events. Typical keywords included extreme, heavy rain, heavy precipitation, etc. The location topic (19.8%) covered the mainly devastated locations where the inner-city flood occurred. These places were primarily the names of the metro station, tunnel, and highway entrance. All of these places had mass casualties occurred. The third topic was the search and rescue efforts (17.1%), including rescue, vehicles, emergency, flood prevention, trapped, escaped, and volunteer. The metro topic (16.8%) was about the failure of the subways, such as the driver, the passengers, and the failure of the subways. Line 5 of the metro system within the city gained the most significant attention from the media and public because 14 persons lost their lives within a truck of the train trapped underground in water. The last topic described the impacts of the hazard (15.4%), being trapped, dead, statistics, perish, stress, etc.

The public comments had different themes in the emergency response period than the media’s posts. Prosocial and help-seeking were the main topics of the public comments. The first topic was help-seeking, which shared about 24% of the contests. We can link the first topic to the fifth one, the places neglected in the media’s report. We also find that these comments initially called for help from residents in rural areas in this region, but mainstream media did not report the disaster situation and the needs of residents in their news reports. As a result, they turned to the official media’s social media accounts to seek help and attention. The second and fourth topics were moral support to the search and rescue workers and condolence to the victims and their families. The donation and charity topic shared 21.7% of the contents and ranked as the number three of the comments from the public.

On January 21, 2022, 6 months after the disaster, an accountability investigation report from the central government gained another peak of attention (Fig. 2). As shown in Table 2, the official media’s posts primarily focused on the contents of the investigation report, and five topics were observed. The first topic was about the attribution of the event and being punished (27.7%). Hiding information, having little risk awareness, and enormous causalities were the primary reasons attributed to the punished local officials.The second one was about the investigation process, which shared 24.8% of the contents. The description of the punishment was the third topic, sharing about 24.2% content, and the keywords were like prosecuted, police, arrested, accountability, criminals, and violated the discipline of CPC. The fourth and fifth topics were about lessons learned from this event. The fourth was primarily about lessons learned for emergency response (14.7%), while the last was about lessons for disaster prevention (8.7%).

**Table 2:** Topics and keywords during the crisis learning period (01/21/2022–03/31/2022).

	Topic	Keywods	%
Media posts	Attribution	People, emergency, event, risk awareness, shortage, hide information, death, missing, exposure, escaped	27.7%
	Investigation	XU Liyi (mayor’s name), CPC secretary, governance council, investigation report, investigation, the national council, punished, filed, State Council of China	24.8%
	Punishment	Henan, police, prosecuted, disciplined, arrested, officials, accountability, criminals, violated the discipline of CPC, related personnel	24.2%
	Response Lessons	People in charge, district/county, leader, response, organizing, distributing, unfulfilled the duty, reflection, flood prevention, housing and recovery	14.7%
	Prevention Lessons	Prevention, works, adaptation, make sure, accountability, responsible work, crisis, arrangement, following order, completely eradicate the risk	8.7%
Public comments	Condolence	Zhengzhou, passed, peace, candle, tear, caring, peaceful, people, death, ritual	24.6%
	Lessons	Mercy, Henan, emergency response, learning from history, harshly punished, lessons learned, natural disaster, understanding, painful	20.8%
	Condemn	Hide information, accountability, fist, governance, resign, lazy, finagle, disappoint, hype, sentimental	20.5%
	Encourage	Add oil, lessons learned, no disaster, accountability, salute, life, Henan, people, huge	18%
	Disaster	Both nature and man-made disaster, death, tear, Zhengzhou, tunnel, natural disaster, rainstorm, hurt, painful, collapse	16.1%

Praise and encourage heroes (18%), condolence to the victims and their families (24.6%), condemnation of the villains (20.5%), and repeating the impacts of the disaster (16.1%) and lessons learned (20.8%) were the five topics that emerged from the public’s comments under the official media’s posts since January 21, 2022 (Table 2).

Compared with the official media posts, which primarily covered the investigation process, the attribution of being punished, the punishment types, and lessons learned from this event, the public’s comments were more emotional, repeated the disaster impact, praised the heroes, expressed condolence to the victims and condemned the variants—the punished local officials and contractors.

Figure 3 compared the differences in emotional expressions between the media posts and public comments. During the emergency response period, the emotion ranking from the official media was liking, anger, disliking, depression, fear, joy, and surprise, while the emotion ranking from the public’s comments was anger, liking, joy, disliking, depression, fear, and surprise. The most significant emotional difference between the media’s posts and the public’s comments was anger and liking.

During the crisis learning period after the accountability investigation report was released, the descending ranking of emotions from official media was depression, liking, disliking, anger, fear, joy, and surprise, while the descending ranking from the public expressions was anger, disliking, liking, depression, fear, joy, and surprise. The proportions of emotions shared between the official media and the public significantly differed between anger and depression. The general public expressed more anger, while the official media’s posts expressed a higher proportion of depression (Fig. 3).

Then we mapped the emotions by topics between the two groups in the two periods (Fig. 4). During the emergency response period, liking was a dominant emotion, and it existed across all five topics and concentrated on the hazard topic and the topic of impacts from the official media’s posts. Anger was the dominant emotion in the public’s comments, while it concentrated on the help-seeking topic. It can be understood that the residents from rural and neglected areas came to social media seeking help, and no wonder they expressed anger in their comments. The joy emotion of the general public was mainly related to the moral support topic.

During the crisis learning period, the official media became very depressed when they mentioned the attribution to the disaster, the lessons learned for disaster response, and the punishment of the local officials in particular. There were some like emotions when the investigation processes were mentioned. For the public, the anger dispersed across all five topics, but it reached a high intensity when the public expressed condolence to the victims and their families. When the disaster impacts were repeated, the expressions demonstrated more depression (Fig. 4).

## Discussion

In this study, we compared the topics and emotions between the official media posts on Weibo and the public’s comments under these posts after the 2021 summer flood, an extreme weather disaster in Henan Province of China. It claimed 398 death, and 380 of them were from the major city Zhengzhou. This analysis can contribute to current knowledge of disaster management and climate change adaptation, as well as the understanding of the communication and behavioral patterns in social media in the following three aspects.

First, we found that the official mass media’s posts on social media platforms primarily focused on the facts of the event and mainly covered the most attractive scenarios in a disaster. The news coverage regarding a disaster is ephemeral, while the marginalized regions impacted can be neglected by mass media. Since only short texts are allowed in social media posts, and the Weibo post has a maximum word limit of 140 characters, the media’s posts should be very concise, and thus they may only cover the facts of an event. The heavy rain and flood impacted a large area in the central region of China. However, the official media primarily focused on the impacts of the big city and infrastructure failure, especially the failure of the subway system and the inundation of the highway tunnel, and little coverage of information regarding the rural areas and small towns flooded during the emergency response period. After the central government released the accountability investigation report, the official mass media’s posts primarily repeated the report’s contents, highlighting the punishment of the local officials and the reasons for being punished. Unlike the preference for antisocial events in disaster-related news in western countries (Rodríguez et al., 2006; Tierney et al., 2006), the reports of disasters in China from the official mass media prefer neutral or positive narratives (Fu et al., 2012; Liu & Boin, 2020; White & Fu, 2012). The ignorance of public attention to rural and remote areas in large-scale disasters is common. They have been observed in previous catastrophes like the 2004 Indian Ocean tsunami (Brown & Minty, 2008), the 2005 hurricane Katrina (Tierney et al., 2006), the 2008 Wenchuan earthquake (Han et al., 2011), and the 2010 Haiti earthquake (Lobb et al., 2012). The media coverage bias can lead to unequal disaster relief distribution, especially when disaster responses heavily rely on the nonprofit sectors or international organizations, like the Haiti earthquake and the Indian Ocean tsunami (Brown & Minty, 2008; Lobb et al., 2012). Therefore, emergency response professionals should include broader intelligence for disaster relief and pay special attention to marginalized groups or areas.

Second, the public’s comments on the official media’s posts on social media demonstrate interesting and important behavioral patterns, especially during the emergency relief period. The official media’s social media accounts have become essential places for people affected by disasters to seek public attention and help, especially for the relatively neglected groups. Besides the expressions of moral support and sympathy to victims in the comments under the media’s posts, most comments were damage reporting and help-seeking from residents in the neglected rural areas. Help-seeking or organized self-help using social media has been observed in many cases (Kaufhold & Reuter, 2016; Silver & Matthews, 2017), especially in the ongoing Covid-19 catastrophe (Chen et al., 2022; Luo et al., 2020). However, previous studies did not report where the victims may come for help. Our finding here has important potential implications for disaster relief in the future. The social media platform, especially the posts under government agencies, official or mass media, and nongovernmental organizations active in disasters, can be a source to identify the needs of the marginalized groups.

Third, the official media’s posts and the public’s comments have some emotional resonance but significantly different topics and concerns. The emotional expressions between the posts from the media and the comments from the public have different emotional patterns—the official media had more positive and depressing emotions. Still, anger was the predominated emotion of the public. Though media narratives can influence public opinion (Chong & Druckman, 2007), our analysis indicated that such effects could be limited. The Weibo platform has its policy regarding displaying or removing content that violates its community standards, such as polarized expression, hate speech, or politically sensitive issues (Zeng et al., 2017). Each Weibo user can also choose to display unwanted comments following their posts. We did find that the number of comments displayed was less than the total number of comments revealed after the posts when we collected the data. Even though we mapped the emotions by topics, we found the prevalence of anger emotion from the general public. Such anger emotion was concentrated on the help-seeking during the emergency response period and the condolence to the victims during the crisis learning period.

Besides the importance of this study aforementioned, this analysis has the following limitations. First, we only investigated the official media posts related to China’s central government agencies. We did not include all the official media, and actually, all the media in China are state-owned. Thus, we cannot discover the different patterns between the media related to central government and local government. Previous analysis revealed that there would be different patterns of narratives between local and national media regarding controversial issues (Du & Han, 2020). Second, since the posts on social media platforms are short, we cannot discover the narrative strategies used in these media. However, since we want to explore the dynamics between the official media and the public, social media is the most suitable place to observe these interactions. Thus, studies using complete news reports from televisions and newspapers can be included to understand the narrative strategies regarding future extreme weather-related disasters.

## Conclusion

The posts from official media related to the central government of China regarding the 2021 summer extreme weather and flood disaster in Henan province and the public’s comments under these posts are analyzed. During the past months from July 2021, when the tragedy occurred, the posts from official media are primarily concentrated in July 2021 and January 2022, when an accountability investigation report regarding this disaster was released. We used the LDA method for topic modeling and the DUTIR for emotion analysis. Overall, the official media’s posts primarily describe the facts and focus on the disaster’s impact on the big city. The public’s comments demonstrate more emotional expressions. During the emergency relief period, the locations, hazards, the infrastructure (metro) failing, the search and rescue, and the impacts are the primary topics from the official media posts. At the same time, the comments from the public concentrate on help-seeking and impacts-reporting from the neglected rural areas, besides emotional expressions like moral support and condolence. When the accountability investigation report was released, the media posts are more about the investigation facts, the punishment to the local officials, and the attribution to the punishment. Lessons learned for disaster prevention, and response are the other two topics from the official media’s posts. The public’s comments primarily cover emotional expressions, such as praising the heroes, condemning the villains, and condolence to the victims, besides the repeated impacts and lessons learned. The emotional expressions between the official media’s posts and the public are relatively coherent but have significantly different patterns. Anger is the dominant emotion of the public in both the crisis response and lesson learning stages. At the same time, depression and liking are the primary emotions from the official media’s posts.

## Supplementary information

The Dalian Polytechnic Emotional Dictionary

## Data Availability

The datasets and code generated and analyzed during the current study are available from the corresponding author upon reasonable request.

## References

[CR1] Abedin B, Babar A (2018). Institutional vs. non-institutional use of social media during emergency response: a case of Twitter in 2014 Australian Bush Fire. Inform Syst Front.

[CR2] Albright EA (2020). Disaster-driven discussion. Nat Clim Chang.

[CR3] Behl S, Rao A, Aggarwal S, Chadha S, Pannu HS (2021). Twitter for disaster relief through sentiment analysis for COVID-19 and natural hazard crises. Int J Disast Risk Reduct.

[CR4] Birkland TA (1997). After disaster: agenda setting. Public Policy, and Focusing Events.

[CR5] Birkland TA (2016) Policy process theory and natural hazards. Oxford Research Encyclopedia of Natural Hazard Science. 10.1093/acrefore/9780199389407.013.75

[CR6] Blei DM, Ng AY, Jordan MI (2003). Latent dirichlet allocation. J Mach Learn Res.

[CR7] Brown PH, Minty JH (2008). Media coverage and charitable giving after the 2004 tsunami. South Econ J.

[CR8] Chen A, Ng A, Xi Y, Hu Y (2022). What makes an online help-seeking message go far during the COVID-19 crisis in mainland China? A multilevel regression analysis. Digit Health.

[CR9] Chen L, Zhang C, Wilson, C (2013) Tweeting under pressure: Analyzing trending topics and evolving word choice on sina weibo. Proceedings of the First ACM Conference on Online Social Networks. Association for Computing Machinery, pp. 89–100

[CR10] Chong D, Druckman JN (2007). Framing theory. Ann Rev Polit Sci.

[CR11] CRED & UNDRR. (2020). The human cost of disasters: An overview of the last 20 years (2000-2019) (p. 30). United Nations Office for Disaster Risk Reduction. https://www.undrr.org/publication/human-cost-disasters-overview-last-20-years-2000-2019

[CR12] Cyberspace Administration of China (2021, October 21) The National Office of the Cyberspace Administration Released the Updated Version of Eligible Entities for Producing and Releasing News on Internet by the Communist Party of China’ Office of Cybersecurity and Information (中国网信网. (2021, October 21). *国家网信办公布最新版《互联网新闻信息稿源单位名单》-中共中央网络安全和信息化委员会办公室*.) http://www.cac.gov.cn/2021-10/18/c_1636153145780775.htm

[CR13] Du Q, Han Z (2020). The framing of nuclear energy in Chinese media discourse: A comparison between national and local newspapers. J Clean Prod.

[CR14] Fang J, Hu J, Shi X, Zhao L (2019). Assessing disaster impacts and response using social media data in China: a case study of 2016 Wuhan rainstorm. Int J Disast Risk Reduct.

[CR15] Fu K, Zhou L, Zhang Q, Chan Y, Burkhart F (2012). Newspaper coverage of emergency response and government responsibility in domestic natural disasters: China-US and within-China comparisons. Health Risk Soc.

[CR16] Haman, M (2020) The use of Twitter by state leaders and its impact on the public during the COVID-19 pandemic. Heliyon, 6(11). 10.1016/j.heliyon.2020.e05540 10.1016/j.heliyon.2020.e05540 PMC7695954 33294685

[CR17] Han Z, Hu X, Nigg J (2011). How does disaster relief works affect the trust in local government? A study of the Wenchuan earthquake. Risk Hazard Crisis Public Policy.

[CR18] Hodson J, Veletsianos G, Houlden S (2021). Public responses to COVID-19 information from the public health office on Twitter and YouTube: implications for research practice. J Inf Technol Polit 1–9. 10.1080/19331681.2021.1945987

[CR19] Hong L, Davison BD (2010) Empirical study of topic modeling in twitter. Proceedings of the First Workshop on Social Media Analytics, Association for Computing Machinery, pp. 80–88

[CR20] Houser M, Gazley B, Reynolds H, Grennan Browning E, Sandweiss E, Shanahan J (2022). Public support for local adaptation policy: The role of social-psychological factors, perceived climatic stimuli, and social structural characteristics. Glob Environ Chang.

[CR21] Howe PD (2021). Extreme weather experience and climate change opinion. Curr Opin Behav Sci.

[CR22] Iglesias-Sánchez PP, Vaccaro Witt GF, Cabrera FE, Jambrino-Maldonado C (2020). The Contagion of Sentiments during the COVID-19 Pandemic Crisis: The Case of Isolation in Spain. Int J Environ Res Public Health.

[CR23] Karimiziarani M, Jafarzadegan K, Abbaszadeh P, Shao W, Moradkhani H (2022). Hazard risk awareness and disaster management: Extracting the information content of twitter data. Sustain Cities Soc.

[CR24] Kaufhold M-A, Reuter C (2016). The self-organization of digital volunteers across social media: the case of the 2013 European floods in Germany. J Homel Secur Emerg Manag.

[CR25] Liu Y, Boin A (2020). Framing a mega-disaster: political rhetoric and the Wenchuan earthquake. Saf Sci.

[CR26] Lobb A, Mock N, Hutchinson PL (2012). Traditional and social media coverage and charitable giving following the 2010 earthquake in Haiti. Prehosp Disast Med.

[CR27] Luo C, Li Y, Chen A, Tang Y (2020). What triggers online help-seeking retransmission during the COVID-19 period? Empirical evidence from Chinese social media. PLoS ONE.

[CR28] Nullis C (2021, October 31) State of climate in 2021: extreme events and major impacts | UNFCCC. https://unfccc.int/news/state-of-climate-in-2021-extreme-events-and-major-impacts

[CR29] Ogunbode CA, Demski C, Capstick SB, Sposato RG (2019). Attribution matters: Revisiting the link between extreme weather experience and climate change mitigation responses. Glob Environ Chang.

[CR30] Peng K-H, Liou L-H, Chang C-S, Lee D-S (2015) Predicting personality traits of Chinese users based on Facebook wall posts. 2015 24th Wireless and Optical Communication Conference (WOCC), 9–14. 10.1109/WOCC.2015.7346106

[CR31] Quercia D, Askham H, Crowcroft J (2012) Tweetlda: Supervised topic classification and link prediction in twitter. Proceedings of the 4th Annual ACM Web Science Conference, Association for Computing Machinery, pp. 247–250

[CR32] Ramage D, Dumais S, Liebling, D (2010) Characterizing microblogs with topic models. Fourth International AAAI Conference on Weblogs and Social Media, Association for Computing Machinery

[CR33] Řehuřek R, Sojka P (2011) Gensim—Statistical semantics in python. Retrieved from Genism. Org

[CR34] Reuter C, Kaufhold M-A (2018). Fifteen years of social media in emergencies: a retrospective review and future directions for crisis Informatics. J Contingencies and Crisis Manag.

[CR35] Ritchie H, Roser M (2022) Natural disasters. Our World in Data. https://ourworldindata.org/natural-disasters

[CR36] Rodríguez H, Trainor J, Quarantelli EL (2006). Rising to the challenges of a catastrophe: the emergent and prosocial behavior following hurricane Katrina. Ann Am Acad Polit Soc Sci.

[CR37] Roxburgh N, Guan D, Shin KJ, Rand W, Managi S, Lovelace R, Meng J (2019). Characterising climate change discourse on social media during extreme weather events. Glob Environ Chang.

[CR38] Saldaña M (2022). Who is to blame? Analysis of government and news media frames during the 2014 earthquake in Chile. Journal Stud.

[CR39] Silver A, Matthews L (2017). The use of Facebook for information seeking, decision support, and self-organization following a significant disaster. Inf Commun Soc.

[CR40] Straub AM (2021). “Natural disasters don’t kill people, governments kill people:” hurricane Maria, Puerto Rico–recreancy, and ‘risk society.’. Nat Hazard.

[CR41] Tierney K, Bevc C, Kuligowski E (2006). Metaphors matter: disaster myths, media frames, and their consequences in Hurricane Katrina. Ann Am Acad Polit Soc Sci.

[CR42] Tsuriel K, Dvir Gvirsman S, Ziv L, Afriat-Aviv H, Ivan L (2021). Servant of two masters: how social media editors balance between mass media logic and social media logic. Journalism.

[CR43] Vo B-KH, Collier N (2013). Twitter emotion analysis in earthquake situations. Int J Comput Linguistics Appl.

[CR44] Waugh WL, Streib G (2006). Collaboration and leadership for effective emergency management. Public Adm Rev.

[CR45] White JD, Fu K-W (2012). Who do you trust? Comparing people-centered communications in disaster situations in the United States and China. J Comparat Policy Anal Res Pract.

[CR46] Wikipedia (2022a) Hurricane Ida. In: Wikipedia. https://en.wikipedia.org/w/index.php?title=Hurricane_Ida&oldid=1085946745

[CR47] Wikipedia (2022b) 2021 European floods. In: Wikipedia. https://en.wikipedia.org/w/index.php?title=2021_European_floods&oldid=1086555248

[CR48] Wikipedia (2022c) 2021 Henan floods. In: Wikipedia. https://en.wikipedia.org/w/index.php?title=2021_Henan_floods&oldid=1086407519

[CR49] Wikipedia (2022d) 2022 Eastern Australia floods. In *Wikipedia*. https://en.wikipedia.org/w/index.php?title=2022_Eastern_Australia_floods&oldid=1086769341

[CR50] Wu D, Cui Y (2018). Disaster early warning and damage assessment analysis using social media data and geo-location information. Decis Support Syst.

[CR51] Wu G, Han Z, Xu W, Gong Y (2018). Mapping individuals’ earthquake preparedness in China. Nat Hazard Earth Syst Sci.

[CR52] Xu L, Lin H, Pan Y, Ren H, Chen J (2008). Constructing the affective lexicon ontology. J China Soc Sci Techn Inf.

[CR53] Zeng J, Chan C, Fu K (2017). How social media construct “Truth” around crisis events: Weibo’s rumor management strategies after the 2015 Tianjin blasts. Policy Internet.

[CR54] Zhang L (2016). Python data analysis and mining practice [M].

