# Supplementary material for: Topical and emotional expressions regarding extreme weather disasters on social media: a comparison of posts from official media and the public
Source: Humanit Soc Sci Commun. 2022 Nov 28;9(1):421. doi: 10.1057/s41599-022-01457-1 (PMC9703438; doi:10.1057/s41599-022-01457-1)
Supplement: Supplementary file 1 — The Dalian Polytechnic Emotional Dictionary [file 41599_2022_1457_MOESM1_ESM.docx]

Topical and emotional expressions regarding extreme weather disaster on social media: a comparison between posts from official media and the public

Ziqiang Han^*^, Mengfan Shen, Hongbing Liu, Yifan Peng

Ziqiang Han, School of Political Science and Public Administration, Shandong University, Center for Crisis Management Research, Tsinghua University, China. Email: ziqiang.han@sdu.edu.cn

Mengfan Shen, School of Information Science and Engineering, Shandong University, Qingdao, China. Email: mengfan@mail.sdu.edu.cn

Hongbing Liu, School of Political Science and Public Administration, Shandong University, Qingdao, China, Email: hongbing.liu@mail.sdu.edu.cn

Yifan Peng, Department of Population Health Sciences, Weill Cornell Medicine, University of Cornell, New York City, USA. Email: yip4002@med.cornell.edu

*** Corresponding author:**

Ziqiang Han, School of Political Science and Public Administration, Shandong University, China. Email: [ziqiang.han@sdu.edu.cn](mailto:ziqiang.han@sdu.edu.cn);


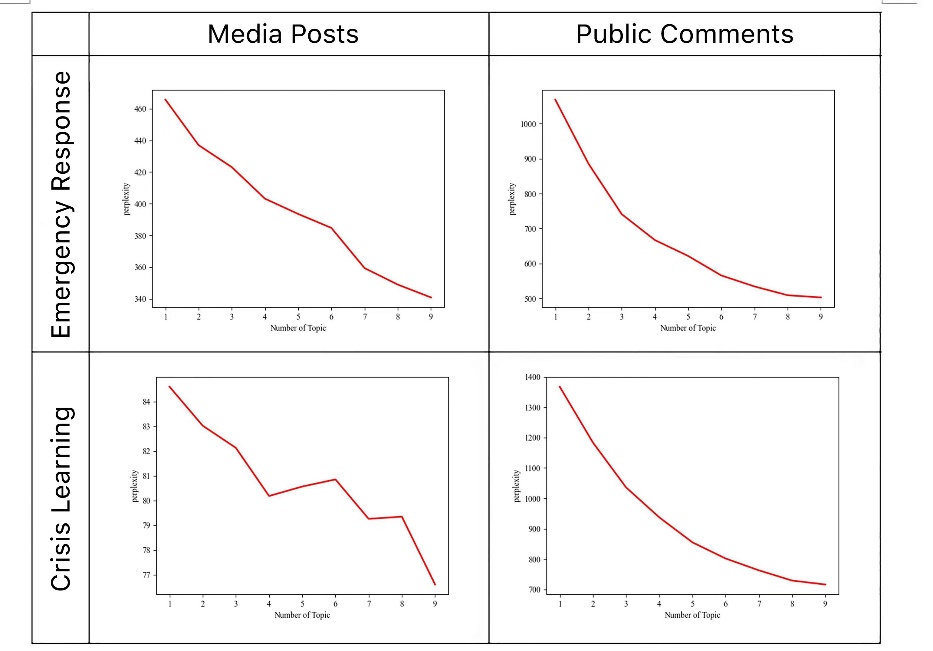


*Fig. S1 Perplexity results according to the number of topics*


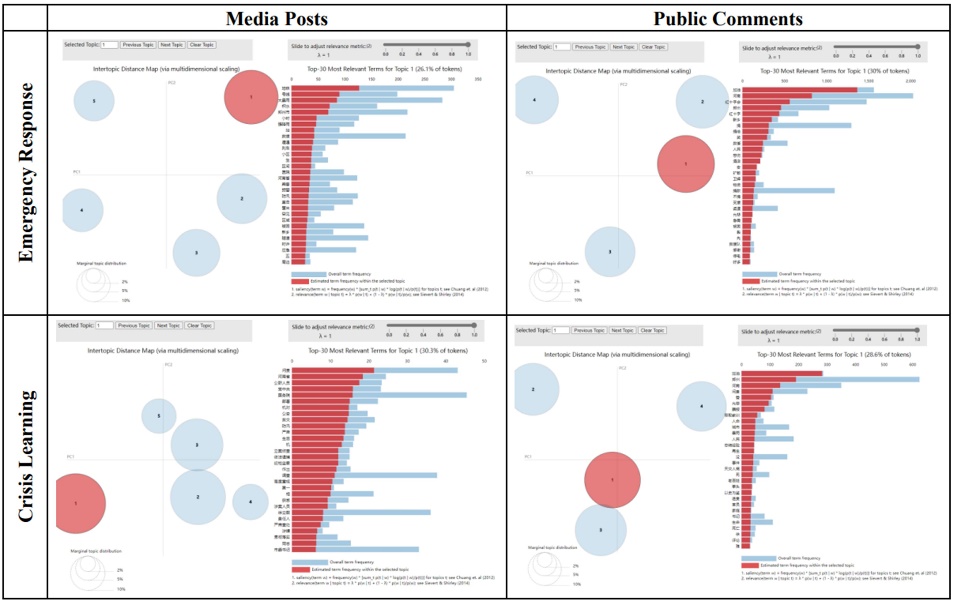


*Fig. S2 Visualized results from the prepare method of pyLDAvis*

Supplementary Table 1: The Dalian Polytechnic Emotional Dictionary

| No. | First level  English/Chinee | Second level  English/Chinese | Positive VS. Negative | Examples (in Chinese) |
| --- | --- | --- | --- | --- |
| 1 | Joy/乐 | Happy/快乐 | Positive-A | 喜悦、欢喜、笑眯眯、欢天喜地 |
| 2 |  | Peace/安心 | Positive-E | 踏实、宽心、定心丸、问心无愧 |
| 3 | Like/好 | Respec/尊敬 | Positive-D | 恭敬、敬爱、毕恭毕敬、肃然起敬 |
| 4 |  | Praise/赞扬 | Positive-H | 英俊、优秀、通情达理、实事求是 |
| 5 |  | Belief/相信 | Positive-G | 信任、信赖、可靠、毋庸置疑 |
| 6 |  | Affection/喜爱 | Positive-B | 倾慕、宝贝、一见钟情、爱不释手 |
| 7 |  | Wish/祝愿 | Positive-K | 渴望、保佑、福寿绵长、万寿无疆 |
| 8 | Anger/怒 | Anger/愤怒 | Negative-A | 气愤、恼火、大发雷霆、七窍生烟 |
| 9 | Depression/哀 | Sadness/悲伤 | Negative-B | 忧伤、悲苦、心如刀割、悲痛欲绝 |
| 10 |  | Disappoint/失望 | Negative-J | 憾事、绝望、灰心丧气、心灰意冷 |
| 11 |  | Guilt/疚 | Negative-H | 内疚、忏悔、过意不去、问心有愧 |
| 12 |  | Miss/思 | Positive-F | 思念、相思、牵肠挂肚、朝思暮想 |
| 13 | Fear/惧 | Panic慌 | Negative-I | 慌张、心慌、不知所措、手忙脚乱 |
| 14 |  | Fear/恐惧 | Negative-C | 胆怯、害怕、担惊受怕、胆颤心惊 |
| 15 |  | Shame/羞 | Negative-G | 害羞、害臊、面红耳赤、无地自容 |
| 16 | Dislike/恶 | Annoy/烦闷 | Negative-E | 憋闷、烦躁、心烦意乱、自寻烦恼 |
| 17 |  | Hate/憎恶 | Negative-D | 反感、可耻、恨之入骨、深恶痛绝 |
| 18 |  | Derogate/贬责 | Negative-N | 呆板、虚荣、杂乱无章、心狠手辣 |
| 19 |  | Jealous/妒忌 | Negative-K | 眼红、吃醋、醋坛子、嫉贤妒能 |
| 20 |  | Doubt/怀疑 | Negative-L | 多心、生疑、将信将疑、疑神疑鬼 |
| 21 | Surprise/惊 | Surprise/惊奇 | Positive-C | 奇怪、奇迹、大吃一惊、瞠目结舌 |
